# Childhood cancer survivors’ struggle for social integration after treatment a mixed-methods exploration of activity and participation

**DOI:** 10.1371/journal.pone.0339084

**Published:** 2026-05-08

**Authors:** Verena Paul, Laura Inhestern, Désirée Sigmund, Jana Winzig, Annette Sander, Stefan Rutkowski, Gabriele Escherich, Corinna Bergelt

**Affiliations:** 1 Department of Medical Psychology, University Medical Center Hamburg-Eppendorf, Germany; 2 German Center for Child and Adolescent Health, partner site Hamburg, Hamburg, Germany; 3 Department of Pediatric Hematology and Oncology, Hannover Medical School, Hannover, Germany; 4 Department of Pediatric Hematology and Oncology, University Medical Center Hamburg-Eppendorf, Germany; 5 Department of Medical Psychology, University Medicine Greifswald, Germany; 6 German Center for Child and Adolescent Health, partner site Rostock/Greifswald, Greifswald, Germany; Touro University California College of Pharmacy, UNITED STATES OF AMERICA

## Abstract

**Purpose:**

Childhood cancer presents lasting challenges beyond primary treatment, affecting multiple aspects of daily life. This mixed-methods study examines the impact of childhood cancer on activities and participation within the first five years after treatment. It mainly focuses on the interplay of factors influencing participation. Further, qualitative interviews provide contextual insights and experiential perspectives from affected families to complement the quantitative findings.

**Methods:**

The study combines qualitative interviews with 30 parents and quantitative survey data from 256 parents. The survey included self-developed items based on the International Classification of Functioning, Disability and Health (ICF). A correlation-based network analysis examined relationships among factors affecting children’s activities and participation.

**Results:**

Survivors experience impairments in school activities, social interactions, and daily functioning. Network analysis highlights the interconnections among ICF-coded aspects, revealing that learning, communication, and participation in major life areas strongly influence overall activity and participation.

**Conclusion:**

While many survivors reintegrate into everyday life, hidden challenges can significantly impact their participation. This study underscores the need for a holistic, multidisciplinary approach addressing medical, educational, and psychosocial challenges, including struggles beyond visible limitations.

**Implications:**

Interventions should prioritize comprehensive support, particularly in learning, communication, and participation in school activity. Educating healthcare providers and caregivers about the interdisciplinary links between these domains is essential for a coordinated, holistic approach.

## Introduction

Childhood cancer exerts a profound impact on the lives of children. Even though the 5-year survival rate for children with cancer has increased to over 80 percent in recent years [1], survivors encounter persistent challenges in everyday functioning beyond the end of treatment [2–5]. Long-term effects can be attributed to the disease itself, as well as to the treatments administered, influencing a child’s psychosocial and cognitive abilities [4,6]. Adolescent and young cancer survivors often express a diminished health-related quality of life [7,8]. Further, prevalent symptoms such as fatigue, sleep disturbances, and pain are frequently reported [9]. Survivors also experience academic difficulties [10], alongside impaired concentration [4,6] and challenges in behavior control [2,11]. They may exhibit inattention to social cues, leading to potential social exclusion by their peers [12]. Although some results indicate that survivors are generally doing well and are thankful [8], the reintegration process does not always go without difficulty. According to an interview study, survivors often perceived that the functional gap significantly changes their daily lives, deviating from their desired expectations [13]. Adolescents expect to regain their pre-cancer level of functionality but face challenges participating in previous activities. However, participation and activity in everyday life activities is essential for children’s physical and mental health. Both is linked to positive academic performance, children’s emotional wellbeing and social behavior [14].

For children returning to everyday life after cancer, careful preparation of families and schools, as well as special support from after-care services, is therefore particularly important. Hence, it is crucial to comprehend the occurrence, interconnections, and consequences of individual challenges after therapy. The International Classification of Functioning, Disability, and Health for Children and Youth (ICF-CY) offers a framework for systematically recording health-related states in children and adolescents [15]. Founded on the biopsychosocial model of health, it reframes functioning not merely as an outcome of disease, but rather as the result of the dynamic interplay between a health issue and contextual factors. The ICF allows for a holistic perspective on health problems and disabilities. It considers not only a person’s medical diagnosis but also the impact on their daily activities and participation in social life. The component “activities and participation” refer to the aspects that outline what a person, dealing with a specific health-related issue, is able or unable to do in their daily life [15].

Using the ICF, Bjorklund et al. demonstrated healthcare professionals predominantly concentrate their documentation on aspects of body function, while information on participation and activities and in everyday life and their inter-correlations are rarely found [16]. This information gap hampers the development of a comprehensive bio-psycho-social understanding of the challenges children face after undergoing cancer treatment. The present study focusses primarily on the participation and activity of childhood cancer survivors (CCS).

### Aims

The primary aim of this study was to examine parent-reported challenges in activity and participation among minor childhood cancer survivors (CCSs) within the first five years after completion of primary treatment, using the ICF framework. Specifically, the quantitative survey data were used to (1) assess parents’ evaluations of their children’s activity and participation based on ICF-related domains, and (2) analyze the interrelationships among individual ICF-based chapters of activity and participation in order to identify key influencing factors. In addition, the qualitative interview data were used to (3) explore how parents perceive and describe their children’s activity and participation in the context of everyday life, thereby providing contextual depth to the quantitative findings.

## Methods

The data was collected as part of a mixed-methods prospective and explorative study, in cooperation with pediatric oncology clinics from northern Germany. Parents completed a paper-pencil-questionnaire with self-generated items on their children’s participation and activity. In addition, we conducted semi-structured interviews with a subset of parents to complement the quantitative survey data by exploring how activity and participation are experienced in everyday life, and to provide contextualized explanations of the survey findings from the parental perspective. Both the items of the questionnaire and the interview questions are based on the ICF-CY. The local ethics committee has approved the study (LPEK-0281). The study protocol was published elsewhere [17] The study is reported following the Consolidated Criteria for Reporting Qualitative Research (COREQ) [18].

### Recruitment

The recruitment took place during the period between 5^th^ of July 2021 and the 31^st^ of December 2023. Parents of minor cancer survivors between 0 and 18 years of age within the first 5 years after the end of acute therapy were eligible to participate. We included both biological and social parents. Furthermore, a signed informed consent form (written) and sufficient knowledge of German were required. Families were either personally informed about the study by the study staff and the healthcare professionals during follow-up appointments or received study information via parents’ self-help organizations or social media groups. The healthcare professionals in the clinics distributed the study materials (questionnaire, information sheet, inform consent form, return envelope). Families who learned about the study through other channels, submitted their contact details via an online contact form. If they wished to participate after receiving all study details, the study materials were sent to them by post. In either recruitment way the consent form was signed and returned to the research team along with the completed questionnaires. The first 98 participants were additionally invited to take part in a qualitative interview, of whom 74 signed and returned the consent form of participation. We recruited a consecutive sample until data saturation was reached, resulting in a final sample of 30 parents for the interview study.

### Quantitative and qualitative data collection

Quantitative data were collected using paper-pencil-questionnaires. Medical information and socio-demographic data were collected via self-developed items. Due to the lack of validated questionnaires, activity and participation were assessed using self-developed items (supplements). Depending on the scope of the ICF-CY chapters within the component “Participation and Activity” of the ICF-CY (*d1: learning and applying knowledge, d2: general tasks and demands, d3: communication, d4: mobility, d5: self-care, d6: domestic-life, d7: interpersonal interaction and relationships, d8 major life areas, d9 community, social and civic life)* [15], the number of developed items varied between a minimum of 2 and a maximum of 5 items per chapter. The questions were introduced with: “Please consider how well your child manages the following areas for their age”, in order to assess parents’ evaluation of limitations in comparison to age-appropriate expectations. The response options were presented on a 5-point Likert scale (1 = never, 2 = rarely, 3 = sometimes, 4 = often, 5 = always). The content of the items was based on the ICF-CY. The item development process followed established procedures described in the literature [15,19]. An example item is shown in Table 1.

**Table 1:** Exemplary item and interview question based on the ICF-CY.

ICF-Code	Questionnaire Items	Interview-questions
Chapter: d1 Learning and applying knowledge		
purposeful sensory experience, watching, listening (d110, d115)	listening or observing attentively	Some parents and teachers report learning/concentration difficulties in affected children at school or at home. How about your child? Does your child have difficulty listening, concentrating on tasks, or completing them? Can you provide examples?
reading (d140), writing (d145), calculating (d150)	For school children: reading writing calculating	
acquiering skills (d155)	learning new skills	
focusing attention (d160)	to concentrate	
solving problems (d175)	understand and solve tasks	

Note: The original survey material was created in German and translated into English by the authors.

Qualitative data were collected using semi-structured interviews. The authors (VP and DS) conducted the interviews via telephone using a semi-structured guideline and audio-recorded them. Both hold degrees in psychology (M.Sc.), possess experience in conducting and evaluating qualitative studies, and were supervised by (LI) (Prof, Ph.D.). At the beginning of the interview, participants were informed about the study background, data protection, and organizational aspects (e.g., audio recording). Subsequently, socio-demographic data and general information about the diagnosis and the treatment were collected. The guide further included questions covering the chapters of the ICF-CY component “Activities and Participation”. An example of a question can be found in Table 1. Parents were consistently encouraged add further relevant issues during the interview. The interview guide underwent pilot testing, with only minor adjustments so that the pilot interview could be incorporated into the analysis. The interviews lasted 57 minutes on average (Range between 40 and 106 min).

### Quantitative analyses

Data preparation and analysis were performed using the statistical program SPSS (version 27, IBM Corporation, 2020). The individual scale scores’ means and standard deviations were calculated for different tumor entities and considering the time since diagnosis. We conducted the mean comparisons using a one-way ANOVA and t-tests.

Further, we employed a graphical model featuring a network based on partial correlations to explore the interrelation of the components of activity and participation [20]. We generated the network using the statistic program JASP. In network analysis, the following metrics provide insights into how nodes (variables) interact within the network. *Betweenness* measures how often a node acts as a bridge or mediator between other nodes. A node with high betweenness lies on many shortest paths between other nodes, playing a central role in connecting different parts of the network. *Closeness* measures how close a node is to all other nodes in the network, based on the shortest paths. A node with high closeness is easily accessible and can quickly influence or interact with other nodes. *Strength* refers to the total weight or number of direct connections a node has with other nodes. A node with high strength has strong and numerous direct connections, indicating its direct influence on the network. *Expected influence* combines both the strength of direct connections and the influence of those connections on other important nodes. It measures a node’s potential to affect the network, both directly and indirectly [21].

### Qualitative analyses

We transcribed the audio recordings verbatim and conducted qualitative structuring content analysis according to Mayring’s approach [22]. Participants did not receive the transcripts for review or correction. In deductively forming the content categories, we aligned with the chapters in the component “Participation and Activity” of the ICF-CY. To establish a coding system, we initially reviewed ten transcripts using the MAXQDA software, conducted by (VP and DS). The coding system underwent consensus-based review and adjustment. Subsequently, a separate portion of the material was double-coded by both interviewers to assess inter-rater reliability. The agreement between coders was 85%. Following this, the coding system underwent further refinement and finalization. Ultimately, (VP) applied the finalized codes to the entirety of the material.

## Results

### Sample characteristics

256 parents (83 fathers, 166 mothers, 2 grandparents and 3 other adult caregivers) from 174 families participated in the quantitative survey. Parents were on average 43 years old (range 28-83y). The CCSs (n = 174) were on average 9 years old (range 1-17y). If assessments from both parents are available for one child, both are included in the analysis.. 30 parents from 28 families participated in the qualitative survey. Of these, 10 were men and 20 were women. The children of the interviewees who survived childhood cancer were on average 9 years old (range 3–17 years) at the time of the interview and 6 years old (range 1–15 years) at the time of diagnosis. 18 of the survivors were male and 10 were female. The survey was conducted on average 2.9 years after diagnosis. Further sample characteristics are displayed in Table 2.

**Table 2:** Sociodemographic data of the participants from the interview study and the quantitative survey.

	Quantitative sample			Qualitative sample		
sociodemographic data (parents)	total (n = 256)	fathers (n = 86)	mothers (n = 170)	total (n = 30)	fathers (n = 10)	mothers (n = 20)
age in years (m, sd)	42.9 (6.7)	45.2 (7.5)	41.7 (6.0)	43 (4.5)	44 (4.7)	42 (4.4)
familial situation						
number of children (m, sd)	2.1 (0,9)	2.2 (1.0)	2.1 (0.7)	2,1 (0.7)	2.0 (0.9)	2,2 (0.5)
permanent relationship (%)	17.8	91.8	91.7	93	100	85
birth country (%)						
Germany	89.3	91.8	88.7	83	90	80
school education (%)						
> 10 years	64.1	11.8	67.5	67	50	55
≤ 10 years	35.9	28.2	32.5	33	50	45
employment status (%)						
full-time	39.2	85.5	13.1	13	40	–
part-time	50.2	10.8	65.5	77	40	85
not employed	8.9	0.2	12.5	7	20	10
parental leave	2.8	0	5.9	3	0	5
other	1.5	2	5	–	–	–
sociodemographic data (cancer survivor)*	total (n = 174)	boys (n = 90)	girls (n = 84)	total (n = 28)	boys (n = 18)	girls (n = 10)
age (m, sd)	9.6 (4.3)	10 (4.3)	9.3 (4.5)	8.6 (3.8)	9.3 (3.8)	7 (3.5)
age at diagnosis (m, sd)	5.7 (4.4)	6 (4.5)	5.4 (4.4)	5.8 (4.1)	6.7 (4.2)	3.9 (3.1)
time since diagnosis (years; m, sd)	2.9 (1.8)	2.8 (1.9)	3.0 (2.3)	3.4 (1.9)	3.1 (1.9)	4 (1.8)
diagnosis* (%)						
leukemia	35	39	30	36	33	40
CNS**-tumor	23	18	28	14	17	10
lymphoma	13	15	10	18	28	0
nephroblastoma	7	7	8	19		20
germ cell tumour	5	5	7	4		10
osteosarcoma	6	6	7	5	11	
soft tissue tumour	4	2	7	5	11	10
others***	11	9	4	4		10
treatment (%)						
chemotherapy	84	91	77	97	95	90
radiotherapy	22	23	21	17	20	10
surgery	62	58	67	47	45	50
stem cell transplantation	7	8	7			

*** melanoma (1); thyroid carcinoma (5); Langerhans cell histiocytosis (6); hepatoblastoma (2) not specified (5)

** CNS, central nervous system.

* 3 patients had two different tumor entities.

### Problems with activity and participation after the end of primary therapy

Descriptive analyses indicate that parents perceive challenges, particularly in general tasks, domestic life, and requirements, as well as in communal, social, and civic life. A comparison between different cancer entities shows that children with brain tumors are perceived to have greater difficulties in learning and applying knowledge, communication, and mobility. No significant differences were observed in the sum scores when considering time since diagnosis. Detailed scale scores can be found in Table 3.

**Table 3:** Activities and participation (mean sum score) of n = 256 CCSs by tumor entity and time since diagnosis.

ICF-classification units	total (256)	leukemia (n = 86)	CNS-tumor (n = 56)	lymphoma (n = 38)	others (74)	≤ 2 years (n = 137)	≥ 2 years (n = 119)
	m(sd)	m(sd)	m(sd)	m(sd)	m(sd)	m(sd)	m(sd)
Learning and applying knowledge (d1)	4.1 (0.7)	4.1 (0.7)	3.7 (0.8)	4.2 (0.7)	4.1 (0.7)	4.1 (0.7)	4.1(0.7)
General tasks and demands (d2)	3.6 (0.8)	3.6 (0.8)	3.4 (0.8)	3.7 (0.8)	3.6 (0.8)	3.5 (0.8)	3.6 (0.8)
Communication (d3)	4.2 (0.7)	4.2 (0.7)	3.8 (0.8)	4.3 (0.5)	4.3 (0.7)	4.2 (0.7)	4.2 (0.6)
Mobility (d4)	4.4 (0.9)	4.5 (0.8)	3.9 (1.1)	4.7 (0.6)	4.7 (0.8)	4.4 (0.9)	4.5 (0.8)
Self-care (d5)	4.3 (0.6)	4.4 (0.5)	4.2 (0.6)	4.3 (0.5)	4.3 (0.6)	4.3 (0.5)	4.2 (0.6)
Domnestic life (d6)	3.7 (1.0)	3.7 (1.0)	3.9 (1.1)	3.5 (1.0)	3.7 (1.0)	3.7 (1.0)	3.7 (1.0)
Interpersonal interactions and relationships (d7)	4.2 (0.5)	4.1 (0.5)	4.0 (0.7)	4.3 (0.4)	4.3 (0.4)	4.1 (0.5)	4.2 (0.5)
Major life areas (d8)	4.0 (0.8)	4.1 (0.8)	3.8 (1.0)	3.9 (0.9)	4.1 (0.9)	3.9 (0.9)	4.0 (0.8)
Community, social and civic life (d9)	3.6 (0.7)	3.6 (0.7)	3.4 (0.9)	3.7 (0.6)	3.8 (0.7)	3.5 (0.7)	3.7 (0.9)

** values that significantly (p < .05) differ from the total mean score are bolded

* values can range from 0 “low activity and participation” to 5 “high activity and participation”

### Interrelations of ICF-Codes

**Fig 1:**
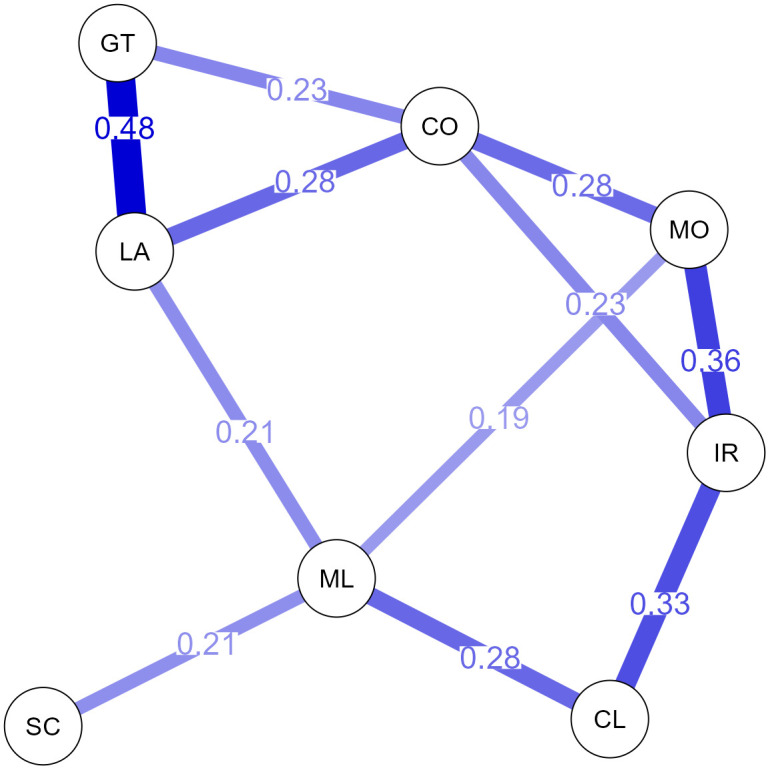
Correlation based network visualization diagram within the ICF-CY component “activity and participation”. ICF- categories with labels (shortened), correlation-coefficient threshold r > .15., No. of chapters = 8, d1 learning and applying knowledge (LA), d2 general tasks and demands (GT), d3 communication (CO), d4 mobility (MO), d5 self-care (SC), d7, interpersonal interactions and relationships (IR), d8 major life areas (ML), d9 community life (CL).

The resulting network (Fig 1) shows a complex structure of components that are interrelated. For a better overview, only connections with a correlation coefficient greater than r=.15 are shown in the final network.

The nodes with the highest betweenness scores were *learning and applying knowledge (d1)* and *major life area (d8)*. These variables serve as critical bridges in the network, connecting other components of participation and activity. The variables with the highest closeness were *major life areas (d9)* and *mobility (d4)*, indicating that these aspects are more easily accessible and have a direct connection to other areas of participation. Mobility demonstrates high accessibility (high closeness) but plays little role as a mediator between other areas. In contrast, *self-care (d5)* and *community life (d9)* showed lower closeness values, suggesting they are less centrally positioned within the network. *Communication (d3)* and *learning and applying knowledge* exhibited the highest strength. *Self-care* and *community life* had lower strength scores, indicating weaker direct connections with other aspects of participation and activity. The variables with the highest expected influence were *communication,* *learning and applying knowledge* and *interpersonal relationships (d7).* These nodes have both strong direct connections and significant influence on other aspects of participation and activity.

### Activity and participation in the context of everyday life

The qualitative interviews offer contextualized insights into how activity and participation are experienced in everyday life by families. Parents reported various deficits in learning and knowledge application that align with the ICF-CY. Survivors encountered difficulties in focusing attention (d160) in school and at home. *„Sometimes he reads a task and then, during the actual work, he no longer has half of it in his mind” (mother of a 13-year-old boy).*

Within the ICF chapter d3 “Communication”, difficulties in non-verbal communication were particularly emphasized as impairing. Although problems with speaking (d330) were reported more frequently, deficits in non-verbal communication (d315) were perceived as more functionally limiting. “*He would like to (express his needs author’s note) but doesn’t know how to do it, and then he becomes quite agitated and sometimes angry*” *(mother of an 11-year-old boy).* Parents have noted difficulties in accurately assessing situations and interpreting the behavior of their peers, particularly among younger children aged 3 to 6 years. This includes challenges in expressing their own feelings and needs. According to the parents, this influenced interpersonal interaction and social relationships (d710). Further, parents observed an “outsider status” of their children. *“Social competence, getting to know new children and fitting in, that is very difficult for him. One of the educators used the word ‘outsider’” (mother of a 6-year-old son).* Parents stated that these deficits lead to mental burden, social withdrawal, and an increase in media consumption.

Younger cancer survivors may take longer to develop autonomy, particularly in toilet training, while older children might still face incontinence or need ongoing assistance with basic care post-therapy (d530). The mobility was more restricted, especially in older children. Survivors experienced a regression in their independence. Mentioned were the use of public transportation, walking longer distances, and riding a bicycle (d450, d460). “*We didn’t want to use public transportation (…) because he didn’t have the strength quite yet” (father of a 15-year-old boy).*

Physical limitations, such as amputations, physical condition issues, and osteoporosis, were more prominent when they hindered participation in school routines or leisure activities (d835, d920). “*He can’t play football, which is difficult for a 12-year-old. He sometimes whines, ‘Mom, why do all say it’s great that you’re healthy now, and I feel worse than during chemo‘“ (mother of a 12-year-old boy).* While some children were fully able to return to their familiar leisure activities, others were only partially able to do so. Others, in contrast, could not return to their old hobbies at all or achieve the same level of participation as their peers. Further, children lack energy (d240) and endurance (d210) for years after completing acute therapy *„She’s just tired and exhausted, and it’s hard to muster the energy“ (mother of a 12-year-old girl).*

Hence, not only leisure activities (d920) but also participation in school activities (d835) were affected. This led to frequent absences, disadvantages in performance assessments, and even delayed school graduations. *“He was incredibly frustrated when he found out that he couldn’t complete his education” (mother of a 17-year-old boy).*

## Discussion

With respect to our first study aim, the results indicate that most children were able to reintegrate into daily life following treatment. However, parents reported ongoing limitations in specific domains of activity and participation. The quantitative data provide an overview of differences across the various tumor entities, but should be interpreted comparatively rather than as absolute indicators of impairment, reflecting parental perceptions relative to age-appropriate expectations. In contrast, the qualitative data offer detailed insights into the specific challenges families experience in everyday life, highlighting nuances that may not be fully captured by the quantitative measures. Restrictions in participation and activity as a consequence of childhood cancer have already been identified in previous studies and were found to be associated with reduced quality of life [23]. Several studies have demonstrated a particularly high risk of long-term impairments in concentration, memory, and physical performance among survivors of brain tumors [23–26]. Our findings are consistent with this finding, as parents of children with brain tumors in our study reported more pronounced challenges in ICF-related domains associated with cognitive and physical functioning.

The second study aim was addressed through the correlation-based network analysis, which explored how different domains of activity and participation are interconnected. The results highlight *learning and applying knowledge* and *major life areas* as central nodes with high betweenness, suggesting that these domains play a key role in linking multiple aspects of participation. Corresponding to the ICF-CY, within this study, *major life areas* primarily encompassed school-related activities and educational participation. The bridging function indicates that difficulties in this domain may be associated with challenges across several other chapters of activity and participation. Consequently, interventions targeting school participation may have the potential to influence a broader range of functional domains. Mobility exhibited high closeness centrality but lower betweenness, indicating that it functions primarily as an enabling resource rather than a mediator between domains. This suggests that mobility facilitates access to social, educational, and everyday activities, which is consistent with previous literature linking mobility limitations to social isolation and reduced participation [27]. It has been repeatedly demonstrated that active engagement in social and sports activities can enhance mobility and strength, contributing to the acquisition of interactive skills [28]. Consequently, CCS face the risk of a mutually reinforcing chain of symptoms. Promoting activity and participation in survivors might be crucial to interrupt the negative spiral of social withdrawal, mobility impairments and academic deficits at an early stage. In contrast, *self-care* and *community life* showed lower closeness values, indicating that these domains are less centrally connected within the network. Lower centrality implies that changes in these areas may have limited spillover effects on other domains. Thus, interventions targeting these domains may need to be more specific and may not automatically lead to broader improvements in activity and participation.

The third aim was addressed through qualitative interviews, which provided insights into how activity and participation challenges manifest in everyday life. Building on the quantitative results, which offered an overview of differences across tumor entities and illustrated the interconnections and interplay of various aspects, the qualitative findings help to contextualize these patterns and show the specific ways in which families experience participation limitations. Parents emphasize deficits, particularly in interpersonal interaction, as well as in participation in school and extracurricular activities. Physical limitations, reduced endurance, and social insecurities were often described as barriers to participation in sports and peer-related activities, sometimes leading to social withdrawal. Prolonged periods of cancer-related isolation may affect children differently depending on their developmental stage. This concerning trend requires immediate attention. Not only because social withdrawal is closely associated with mental health [29,30], but also because interaction with peers has a significant impact on the development of children and adolescents. While younger children may be particularly vulnerable due to reduced opportunities to acquire and practice foundational social skills [31], older children and adolescents may experience cancer-related isolation as especially challenging because peer relationships become increasingly central for social belonging, identity formation, and emotional support [27,29,30]. Participation in social and school activities should therefore be actively encouraged and supported. The results raise the question of whether early interventions targeting social skill development, emotional expression, and structured peer interaction during the acute phase of cancer treatment could play a role in reducing social isolation and preventing delays in social competencies. Regarding academic functioning, parents generally reported no or only minor difficulties in basic skills such as reading, writing, or arithmetic. This contrasts with previous studies reporting academic impairments, particularly among survivors [32–34]. One possible explanation for this discrepancy is that parent reports may capture functional difficulties in everyday school life rather than formal academic outcomes. Difficulties in attention, executive functioning, or coping with school demands have been described in childhood cancer survivors across diagnostic groups and may affect daily classroom functioning without necessarily resulting in immediate deficits in basic academic skills [6]. Particularly during or shortly after treatment, children may still compensate for academic content while subtle cognitive or psychosocial challenges become more salient to parents. Consequently, parents may report fewer scholastic impairments but more pronounced school-related functional difficulties.

### Implications

The results of this study highlight specific areas of activity and participation in which childhood cancer survivors face challenges during the first five years after primary treatment. These findings underscore the importance of early, continuous assessment and monitoring of activity and participation, alongside physical, cognitive, and psychosocial functioning. The interconnected nature of these challenges indicates that follow-up care should adopt a holistic and interdisciplinary approach, rather than focusing solely on isolated symptoms.

Early and continuous assessment is crucial, and interventions such as adaptive teaching, occupational therapy, and structured peer- or social-skill programs can help mitigate limitations and prevent a negative spiral of social withdrawal, physical constraints, and academic difficulties.

Successful reintegration requires close collaboration among families, healthcare providers, educational staff, and community organizations, ensuring that interventions are coordinated across medical, psychosocial, and educational contexts. Raising awareness about the potential challenges of childhood cancer among teachers, peers, and the wider community is also essential for creating an inclusive environment that supports full participation and developmental needs.

### Limitation

Some limitations must be taken into account when interpreting the results. The study was conducted exclusively in German, which means that difficulties experienced by patients and families with language barriers can only be depicted to a limited extent. In addition, we used self-generated items. Although these are based are developed based on the ICF-CY, they were not psychometrically evaluated. In some cases, reports from both parents of a single child were included in the network analysis, which may have introduced dependency in the data and could slightly overrepresent certain perspectives. Further, the strength of correlation-based networks lies in their graphical representation of the interconnectedness of codes. Nevertheless, information about the context in which the codes occur together is lost. We have tried to counteract this with the interviews. Consequently, the conclusions drawn from the network are constrained and may not be fully reproducible in different contexts. It is important to note that the interview questions were designed to be semi-structured and open-ended and do not directly align with the questions in the questionnaires.

## Conclusion

Our study focused on exploring different facets of participation and activity experiences following cancer treatment in children. We explored the challenges and consequences, including physical, mental, and social deficits, influencing their participation in daily activities. Brain tumor patients are particularly restricted in some areas. The potential interplay of symptoms highlights the importance of promoting activity and participation while considering long-term effects. Doing so can help disrupt negative spirals, such as physical inactivity leading to decreased fitness or social isolation resulting in anxiety and depression. A particular influence appears to be the ability of learning and applying knowledge as well as participation in major live areas such as school and school-related activities. Additionally, a comprehensive aftercare approach involves addressing broader aspects beyond individual symptoms, recognizing the interconnected nature of the challenges in reintegration into everyday life faced by CCS.

## Supporting information

S1 Table 1 Linkages of the ICF codes with the questionnaire items and the interview guide. (PDF)
