# Supplementary material for: Childhood cancer survivors’ struggle for social integration after treatment a mixed-methods exploration of activity and participation
Source: PLoS One. 2026 May 8;21(5):e0339084. doi: 10.1371/journal.pone.0339084 (PMC13155590; doi:10.1371/journal.pone.0339084)
Supplement: S1 Table — (PDF) [file pone.0339084.s001.pdf]

# S1 Table

S1 Table 1 Linkages of the ICF codes with the questionnaire items and the interview guide.

| ICF-Code | Questionnaire Items | Interview-questions |
|---|---|---|
| ***Chapter d1: learning and applying knowledge*** | | |
| Purposeful sensory experience, watching, listening (d110, d115) | Listening or observing attentively | Some parents and teachers report learning/concentration difficulties in affected children at school or at home. How about your child?<br>Does your child have difficulty listening, concentrating on tasks, or completing them? Can you provide examples? |
| Reading (d140), writing (d145), calculating (d150) | For school children: reading writing calculating | |
| Acquiering skills (d155) | Learning new skills | |
| Focusing attention (d160) | To concentrate? | |
| Solving problems (d175) | Understand and solve tasks? | |
| ***Chapter d2: general tasks and demands*** | | |
| Undertaking a single task (d210) | demonstrate perseverance | Does your child have difficulties completing a task? Can you provide examples?<br>To what extent did your child cope with the tasks and demands at school/daycare after overcoming cancer?<br>What do you think, does your child find it harder or easier compared to peers (e.g., dealing with difficult situations)? |
| Undertaking multiple tasks (d220) | Processing several things at the same time (e.g. listening and taking notes) | |
| Handling stress and other psychological demands (d240) | Being able to withstand stress | |
| Control his/her behaviour (d250) | Thinks before acting | |
| ***Chapter d3: communication*** | | |
| Communicating - producing (d330, d335) | to make clear what he/she wants to say | Do you sometimes find it difficult to understand your child?<br>Does your child have difficulties expressing themselves in a way that others can understand the content they want to convey?<br>If yes, can you provide an example? |
| Communicating with - receiving - nonverbal messages (d315) | …to be able to assess and evaluate social situations correctly | |
| ***Chapter d4: mobility*** | | |
| walking (d450) | walking around independently | Thinking about physical activities, does your child sometimes have problems keeping up with others (e.g. in sports lessons, leisure activities)?<br>If so, can you describe these problems in more detail? |
| Moving around in different locations (d460) | …Perform automated movement sequences (e.g. running, jumping, cycling) | |
| ***Chapter d:5 self-care*** | | |
| Looking after one's health (d570) | …Pay attention to a healthy lifestyle (e.g. diet, sport, media consumption) | Have you experienced or are you experiencing particular challenges in your everyday life due to your child's cancer (taking medication, diet)? |
| Dressing (d540), eating (d550), drinking (d560) | ...my child coped well with everyday life (eating, drinking, dressing) | |

| | | |
|---|---|---|
| | | If yes: Does this result in any restrictions or burdens? |
| ***Chapter d6: domnestic life*** | | |
| Assisting others (d660) | Assisting others | |
| Doing housework (d640) | generally obedient | |
| ***Chapter d7: interpersonal interactions and relationships*** | | |
| basic interpersonal interactions d710 | Understand the feelings and thoughts of others | Do you feel that he struggles more than other children to initiate contact and establish or maintain friendships? |
| Family relationships d760 | ...my child got along well with us as parents | When you think about your family life, has it changed compared to the time before the diagnosis? If so, in what way? (e.g. division of roles or sibling relationships) |
| Informal social relationships (d750) | ...my child is "well received" by others | |
| ***Chapter d8: mahor life areas*** | | |
| prescool education d815 school education d820, | ...my child missed something at (pre)school because of the illness | How was the school situation/situation in kindergarten after the end of intensive therapy? |
| School life and related activities d835 | ...having energy for kindergarten/school | What does it look like today? |
| ***Chapter d9: community, social and civic life*** | | |
| Recreation and leisure d920 | ...my child laughed a lot and had fun | Have your child's leisure activities changed as a result of the illness? |
| Community life d910 | my child has done something together with friends | If yes, what changed? |
